# Molecular signature of methotrexate response among rheumatoid arthritis patients

**DOI:** 10.3389/fmed.2023.1146353

**Published:** 2023-03-27

**Authors:** Boel Brynedal, Niyaz Yoosuf, Tinna Bjorg Ulfarsdottir, Daniel Ziemek, Mateusz Maciejewski, Lasse Folkersen, Helga Westerlind, Malin Müller, Peter Sahlström, Scott A. Jelinsky, Aase Hensvold, Leonid Padyukov, Nancy Vivar Pomiano, Anca Catrina, Lars Klareskog, Louise Berg

**Affiliations:** ^1^Translational Epidemiology, Institute of Environmental Medicine, Karolinska Institutet, Stockholm, Sweden; ^2^Centre for Epidemiology and Community Medicine, Region Stockholm, Stockholm, Sweden; ^3^Division of Rheumatology, Department of Medicine Solna, Karolinska Institutet and Karolinska University Hospital, Stockholm, Sweden; ^4^Center for Molecular Medicine, Karolinska Institutet, Stockholm, Sweden; ^5^Pfizer, Cambridge, MA, United States; ^6^Nucleus Genomics, New York, NY, United States; ^7^Clinical Epidemiology Division, Department of Medicine Solna, Karolinska Institutet, Stockholm, Sweden; ^8^Center for Rheumatology, Academic Specialist Center, Region Stockholm, Stockholm, Sweden

**Keywords:** rheumatoid anhritis, treatment response, methotrexate, gene expression, flow cytometry, plasma proteins, transcriptomics

## Abstract

**Background:**

Methotrexate (MTX) is the first line treatment for rheumatoid arthritis (RA), but failure of satisfying treatment response occurs in a significant proportion of patients. Here we present a longitudinal multi-omics study aimed at detecting molecular and cellular processes in peripheral blood associated with a successful methotrexate treatment of rheumatoid arthritis.

**Methods:**

Eighty newly diagnosed patients with RA underwent clinical assessment and donated blood before initiation of MTX, and 3 months into treatment. Flow cytometry was used to describe cell types and presence of activation markers in peripheral blood, the expression of 51 proteins was measured in serum or plasma, and RNA sequencing was performed in peripheral blood mononuclear cells (PBMC). Response to treatment after 3 months was determined using the EULAR response criteria. We assessed the changes in biological phenotypes during treatment, and whether these changes differed between responders and non-responders with regression analysis. By using measurements from baseline, we also tried to find biomarkers of future MTX response or, alternatively, to predict MTX response.

**Results:**

Among the MTX responders, (Good or Moderate according to EULAR treatment response classification, *n* = 60, 75%), we observed changes in 29 partly overlapping cell types proportions, levels of 13 proteins and expression of 38 genes during treatment. These changes were in most cases suppressions that were stronger among responders compared to non-responders. Within responders to treatment, we observed a suppression of FOXP3 gene expression, reduction of immunoglobulin gene expression and suppression of genes involved in cell proliferation. The proportion of many HLA-DR expressing T-cell populations were suppressed in all patients irrespective of clinical response, and the proportion of many IL21R+ T-cells were reduced exclusively in non-responders. Using only the baseline measurements we could not detect any biomarkers or prediction models that could predict response to MTX.

**Conclusion:**

We conclude that a deep molecular and cellular phenotyping of peripheral blood cells in RA patients treated with methotrexate can reveal previously not recognized differences between responders and non-responders during 3 months of treatment with MTX. This may contribute to the understanding of MTX mode of action and explain non-responsiveness to MTX therapy.

## Introduction

Rheumatoid arthritis (RA) is a chronic inflammatory disease caused by genetic and environmental factors, resulting in symmetric inflammation and destruction of the joints (1). First-line treatment for RA is methotrexate (MTX). Treatment with MTX leads to suppression of immune cells, for example decreased cytokine production by T-cells (2). Tasaki et al. has shown that successful drug treatments (MTX, infliximab or tocilizumab in different individuals) alter the molecular profile closer to that of healthy controls at the transcriptome, serum proteome, and immunophenotype level (3). In their paper the effect of MTX was smaller than the effect of other treatments, but the specific effects of MTX were not elucidated and the number of patients on MTX was small (ten responders and 11 non-responders). It is still unknown which effects of MTX that specifically ameliorates RA symptoms. Between 20 and 40% of RA patients do not respond to MTX, and it is known that response to first-line treatment predicts long-term outcomes in RA patients (4). It would therefore be valuable to understand how the biological effect of MTX differs between responders and non-responders. Such knowledge may provide insights into which mechanisms that can be regulated in order to avoid disease progression.

To investigate the effect of treatment, a good classification of treatment response is needed as well as relevant biological measurements. Patients with RA are routinely examined for their level of inflammation, number of inflamed joints and overall assessment of health. From these measurements the disease activity score DAS28 is calculated (5). In this prospective project, we evaluated the EULAR response criterion after 3 month of MTX in DMARD monotherapy (6). We investigated a wide range of potential biomarkers measured in peripheral blood before treatment initiation and after 3 months of MTX treatment. Gene expression was measured by RNA sequencing; absolute cell counts, cell proportions and phenotypes was measured by flow cytometry; protein levels were measured in serum or plasma. These measurements reflect biological processes within the individual, and we hypothesized that a subset of such measurements may be suitable as biomarkers for the responsiveness to treatment. We also had information regarding several factors known to impact treatment response, such as smoking status, age, sex, and steroid treatment, in the newly diagnosed RA patients before staring MTX treatment.

Our primary aim was to investigate the biological effect of MTX among RA patients, and whether these effects differed between responders and non-responders. Secondarily, we also investigated if we could predict MTX response based on cellular, molecular and clinical features at baseline.

## Materials and methods

### COMBINE cohort

We utilized the COMBINE cohort, which includes 246 individuals, whereof 92 are treatment naïve early RA patients who started MTX treatment at Karolinska University Hospital with a maximum symptom duration of approximately 14 months before inclusion to the study. Demographics and clinical phenotypes at baseline are shown in Table 1. Patients donated peripheral blood at the appointment prior to MTX initiation. All patients returned for a follow up visit after approximately 3 months (full range 67–126 days, median 93 days) where they again underwent a clinical examination and donated peripheral blood (stored in −80°C). Out of the 92 patients who started MTX therapy 12 dropped out during the follow-up period, leaving a total of 80 patients for which we have biological measurements and clinical follow up data (Table 1). The majority of patients in our cohort were prescribed Prednisolone treatment (Table 1), a glucocorticoid with immune suppressive effects. Prednisolone was prescribed either before initiating MTX treatment (N:19) or along with MTX prescription (N:25).

**Table 1:** Demographics at inclusion of the 80 patients with newly diagnosed RA during 2011–2013 who consented to participation and contributed blood at both time points.

	RA (*n* = 80)
Female (%)	60 (75.0)
Age, median years (range)	62 (22–88)
HLA-DR shared epitope positive (%)	54 (67.5)
Self-reported Swedish ethnicity (%)	66 (82.5)
Current smoker (%)	27 (34.6)
Symptom duration in days, median (range)	168 (46–428)
Erosions/osteopenia (%)	20 (25)
ACPA positive (%)	49 (61.3)
DAS28, median (range)	5.0 (0.84–7.6)
Patient global health assessment, median (range)	47 (1–100)
Health professional global health assessment, median (range)	45 (2–84)
Physical function (HAQ), median (range)	1.06 (0–2.5)
CRP, median (range)	6 (0.5–146)
Prednisolone treatment (%)	44 (55.0)

Patients were asked about their ethnicity as well as past and current smoking behavior. We detected anti-citrullinated protein autoantibodies (ACPA) using the multiplex anti-CCP2 assay (Eurodiagnostica) at Karolinska University Hospital. The presence of joint erosions or bone decalcifications was detected using X-ray and assessed by radiologists at Karolinska University Hospital.

### Response outcome

The primary outcome was determined using the EULAR response criteria (7). We dichotomized response, so that those who achieved Good or Moderate EULAR response at 3 months of treatment were considered “responders” and the rest being “non-responders.”

### Flow cytometry

The Clinical Chemistry laboratory of Karolinska University hospital measured the concentration of leukocytes, neutrophils, eosinophils, basophils and monocytes per liter of peripheral blood using XE Sysmex flow cytometry-based analysis.

Additionally, several immune cell phenotypes were measured by flow cytometry at the Rheumatology Laboratory at the Center for Molecular Medicine, Karolinska Institutet (for an overview of the gating strategy see Supplementary Figure S1). Peripheral blood mononuclear cells (PBMC) were isolated and whole blood lysed using Serotec Erythrolyse buffer (Bio-Rad AbD Serotec Ltd). Cells were stained freshly using the following antibodies (clones): CD45RA (B56), TcRgd (B1), HLA-DR (L43), CD4 (OKT4), CD138 (ID4 or DL-101), CD19 (HIB19), NKp44 (P44-8), CD16 (3G8), CD69 (FN50), CD28 (CD28.2), CD45 (HI30), IL21R (2G1-K12), TREM-1 (TREM-26) all from Biolegend, CD3 (UCHT1) and NGG2A (Z199.1) from Beckman Coulter, IgD (IA6-2), CD14 (Mphi 9), CD27 (M-T271), CD56 (BI59) from Beckton Dickinson, NKG2D (1D11) from eBioscience. Only the HLA-DR staining was controlled using an isotype control antibody from Biolegend, while the staining of NKG2A, NKG2D, IL21R and TREM-1 were controlled by absence of added antibody (FMO, fluorescence minus one). The stainings were performed using different antibody panels. One panel focusing on T-cell stainings of PBMC, another on B-cell stainings, a third on NK cells and monocytes, and a fourth staining performed on whole lysed blood where granulocytes were identified by size and granularity (forward and side scattering properties). All measurements were performed on Gallios flow cytometer (Beckman Coulter) and data analyzed using FlowJo (TreeStar Inc., Ashland, OR, United States). In the statistical analyses we utilized a total of 427 flow cytometry variables.

### Protein measurements

We collected information of plasma protein concentrations using different multiplex platforms as described previously (8). Protein levels below detection level were re-coded as 0.001. Within the cohort the distributions of plasma protein concentrations were highly skewed with long tails, and a log transformation was therefore applied prior to association analysis. Only when at least 8 different protein levels above detection threshold within each test was available the proteins were considered for further analyses, resulting in 51 analyzed proteins, including 16 proteins measured using multiple methods.

### RNA sequencing

RNA was purified from PBMCs and sequenced as previously described (8). After removing samples with insufficient quality, we obtained 60 high quality RNA seq samples from the baseline visit, and 60 RNA samples from the follow up visit. From 52 patients we obtained a high-quality RNA seq data set from both baseline and follow up. For 30 of the samples in the MTX cohort the initial sequencing produced very few reads and was therefore repeated. The read files from the two sequencing rounds were merged. We employed Trimgalore (v. 0.4.1) to remove adapter sequence and low-quality bases from reads (−-paired --phred33 --length 25), and reads were aligned to the human genome using STAR (v. 2.5.3a) and summarized across genes using the *gencode* (v.27) annotation. The alignments were performed on resources provided by the SNIC informatics network through Uppsala Multidisciplinary Center for Advanced Computational Science (UPPMAX).

### Statistical analysis

We performed both longitudinal and cross-sectional analyses for each biological measurement. The aim of the longitudinal analyses was to identify changes between baseline and the 3 months visit for responders and non-responders separately, and to analyze whether there were any differences in changes between these two groups. The measurements at baseline were also used to investigate whether any features seen at baseline could predict response after 3 months. All analyses were performed in R (v.3.5.1), and gene expression analyses using DESeq2 (v. 1.20.0).

When analyzing the change in protein and flow cytometry measures during treatment we used a mixed linear model (lme from nlme v. 3.1–137) and assessed the different contrasts using *emmeans* (*lsmeans* v. 2.30–0). We modelled each measurement as dependent on time point (baseline or follow-up), response, an interaction between time point and response, prednisolone, and a random effect of each individual.

When modelling gene expression changes during treatment we wanted to model changes both within (treatment effect) and between (responders vs. non-responders) samples obtained at baseline and at 3 months, respectively. We used the approach outlined in *edgeR* user guide regarding comparisons both between and within subjects (9). Further, gene expression is known to vary greatly between and within individuals, and a major part of this variation is due to differences in proportion of different cell populations in peripheral blood. In our gene expression analyses we therefore aimed to describe changes that are not due to changes in major cell type proportions, but due to changes in gene expression within cells. Changes in cell composition, on the other hand, are better detected using flow cytometry data. We therefore chose to adjust our analysis of gene expression levels based on the proportion of major immune cell types in PBMCs: B-cells, T-cells, NK-cells, or monocytes out of total PBMC. Gene expression was thereby modelled as dependent on response, the interaction between response and patient ID, the interaction between visit and response, prednisolone, the proportion of B, T, NK-cells, and the proportion of monocytes.

In all the prospective analyses using baseline data (gene expression, protein levels, or cell type proportions) to detect biomarkers of response to MTX after 3 months, we adjusted our analyses for factors that might be associated to both response status and biomarker levels (i.e., likely confounders). All baseline analyses were accordingly adjusted for age, sex, whether the individual was of self-reported Swedish ethnicity, had erosions at baseline, was a current smoker when treatment was initiated, presence of ACPA, and treatment with Prednisolone at the time of blood donation. For flow cytometry and measurement of plasma protein concentrations, we analyzed the association between response status and cellular or protein phenotypes using logistic regression.

A few additional covariates were included in the cross-sectional analysis of gene expression data at baseline. We used principal component analysis (PCA) of variance stabilized gene expression data (*rlog* in DESeq2) to look for outlier RNA seq samples. PCA revealed no outlier samples, nor any separation between baseline visit and follow up visit or responders and non-responders (data not shown). PCA analysis revealed a major axis of variation that was strongly, but not completely, associated to measured RNA quality scores (*r*^2^: 0.60). We estimated a surrogate variable (using *svaseq* v. 3.28.0) in the baseline sample set to account for this major axis of variation, which was included as a covariate in cross-sectional gene expression modelling. Since the major cell type proportions of PBMCs are likely confounders, they were again included as covariates in the DESeq2 analysis.

We experienced that DESeq2 was sensitive to single high-count outliers in the cross-sectional analyses, and we therefore implemented a leave-one-out (LOO) approach to assess the stability of the detected gene expression biomarkers. In each iteration, one sample was excluded, and the cross-sectional analyses repeated.

In all gene expression association analyses, we chose to analyze genes where at least 20% of the samples has a normalized count of one or higher, and we did not shrink the log2 (fold changes). Significance was assessed using a Wald test.

Gene set enrichment was investigated using a non-parametric test on gene ranks (tmodCERNOtest function in *tmod* (v. 0.36)), using 1329 canonical pathways (8904 genes) from KEGG,^1^ BioCarta,^2^ Signal Transduction KE,^3^ SigmaAldrich,^4^ Signalling Gateway,^5^ SuperArray SABiosciences,^6^ Pathway Interaction Database,^7^ reactome^8^ and Matrisome Project,^9^ collected by MSigDB. We tested whether gene sets were enriched for having smaller probability values, higher fold change and lower fold change. To avoid enrichments due to lowly expressed genes with inflated fold changes we only report those gene sets that showed significant enrichment both in probability values and fold change ranking.

For all tests we defined a false discovery rate (FDR) (10) of <10% as significant. Each analysis type and biological data type was evaluated separately.

### Prediction

Prediction models were built based on measurements in treatment naïve individuals and based on the difference between post-treatment and pre-treatment measurement.

Three methods were used to classify the response data: a linear method (regression with L1 and L2 regularization *via* the *glmnet* R library), a non-linear method (*via* the *randomForest* library in R), and a kernel-based method (SVM with an RBF kernel, *via* the *smvRadial* library in R). Each learning task was performed in ten repeats, with five-fold cross-validation and with 100 randomly sampled steps of hyperparameter estimation. Covariates outlined above were included as features in each run, and we built predictive models based on gene expression, flow cytometry, protein levels and clinical data, separately and in an integrated fashion. We removed all zero count genes from the expression data, and filtered ncRNAs (miRNA, piRNA, rRNA, siRNA, snoRNA, and tRNAs). In addition, pseudogenes that are lowly expressed and showed high variance were not used in the model. We used protein-coding genes and long non-coding genes in the expression matrix, which resulted in a total of 22,628 genes. The filtered gene expression matrix was normalized using transcript-per-million (TPM).

The performance of resulting models was reported using balanced accuracy and receiver operating characteristic (ROC) curves. Balanced accuracy and area under the ROC curves (AUCs) are calculated as the mean and 95% confidence intervals for each of the repeats in each task. For each ML task, we report the results from the repeat displaying the median of the mean ROC AUCs.

## Results

### Effects of MTX treatment

#### Clinical effects of MTX treatment

Out of the 80 patients, 32 experienced a good EULAR response, 28 a moderate response and 20 were non-responders according to EULAR response criteria. As expected, MTX treatment had a significant ameliorating effect across clinical parameters for responders, while the effect was lower among the non-responders (see Supplementary Table S1).

#### Changes in cell concentrations and phenotypes during MTX treatment

**Figure 1:**
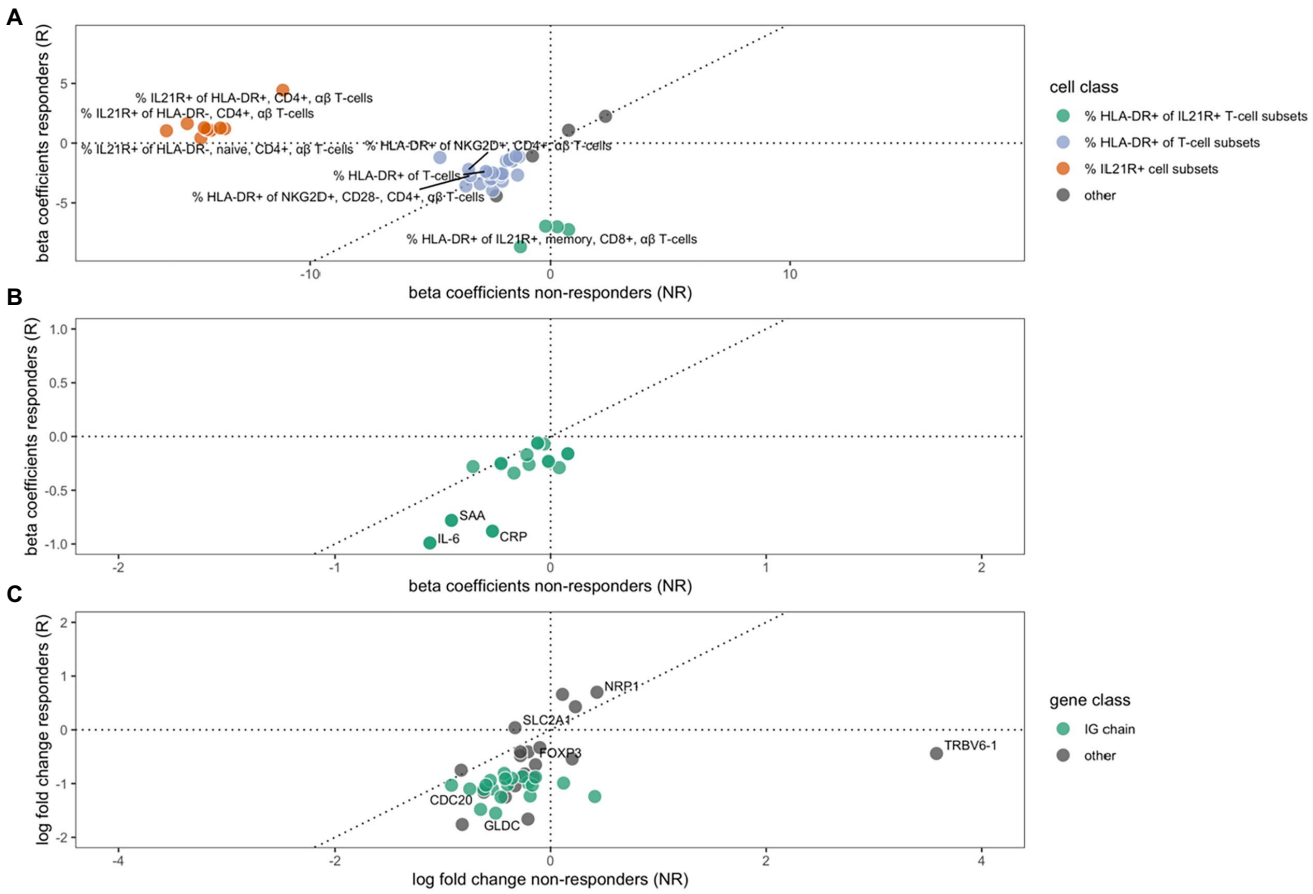
The effect of MTX treatment in non-responders (x-axes) or responders (y-axes) on cell type phenotypes **(A)**, protein expression **(B)** and gene expression **(C)**. Beta coefficients in **A** and **B**, and log_2_ (fold changes) in **C**, of variables that are significantly regulated (FDR *≤* 10%) during MTX treatment in either non-responders, responders, or both. A dotted 45° inclined line indicates the expected parity for individual effects for groups of non-responders and responders to MTX. **(A)** The changes in cell type phenotypes as measured by flow cytometry. Three different groups of cell phenotypes are indicated by colors: The proportions of HLA-DR+ out of different IL21R+ CD4- T-cell subsets (green), the proportion of HLA-DR+ out of different T-cell subsets (purple), and the proportion of IL21R+ out of several T-cell subsets (red). **(B)** The changes in protein expression. **(C)** The change in gene expression, where a group of immunoglobulin genes are indicated by green color.

We analyzed cell concentrations and proportions of cellular phenotypes among responders and non-responders to MTX. A total of 29 flow cytometry measurements were altered during treatment in responders, and 15 in non-responders (Figure 1A), of which only three were shared in both groups (Table 2). These three all mark a similar decrease of the proportion of HLA-DR-expressing T-cells (HLA-DR+ T-cells, HLA-DR + NKG2D + CD4 + gd- T-cells, and HLA-DR+ CD28 + CD4 + gd- T-cells) among responders and non-responders. Overall, the changes in cell proportions within responders during treatment are dominated by a reduction of the proportions of different HLA-DR+ T-cell subsets. We note that the proportion of several HLA-DR+ subsets of IL21R + CD4- T-cells were strongly suppressed among the MTX-responders, while not affected among non-responders (marked by green in Figure 1A). Among the non-responders, we instead noticed a very strong reduction in the proportion of IL21R+ T-cell subsets (Figure 1A).

**Table 2:** The regulation of cell proportions and within patients during MTX follow up.

	Change in non-responders		Change in responders		Difference	
Flow cytometry measure	Beta	FDR	Beta	FDR	Beta	FDR
% HLA-DR+ cells of IL21R+ CD45RA− CD4-gd- T-cells	−1.26	0.88	−8.67	4.2E-03	−7.41	0.37
% HLA-DR+ cells of IL21R+ CD28− CD4-gd- T-cells	0.75	0.94	−7.24	0.023	−7.99	0.35
% HLA-DR+ cells of IL21R+ CD4-gd- T-cells	0.28	0.99	−7.00	0.013	−7.28	0.33
% HLA-DR+ cells of IL21R+ CD28+ CD4-gd- T-cells	−0.21	0.99	−6.95	0.020	−6.74	0.42
MFI NKG2A of NKp44+ CD16- NK cells	−2.27	0.60	−4.41	0.020	−2.15	0.88
% HLA-DR+ cells of NKG2D+ CD45RA− CD4-gd- T-cells	−2.42	0.33	−3.96	3.9E-03	−1.54	0.88
% HLA-DR+ cells of NKG2D+ CD45RA− CD4+ gd- T-cells	−3.54	0.12	−3.58	3.9E-03	−0.04	0.99
% HLA-DR+ cells of CD45RA− CD4− gd- T-cells	−2.94	0.27	−3.40	0.010	−0.47	0.99
% HLA-DR+ cells of NKG2D+ CD28+ CD4-gd- T-cells	−2.02	0.33	−3.18	3.9E-03	−1.16	0.88
% HLA-DR+ cells of CD28+ CD4- gd- T-cells	−2.52	0.27	−3.08	7.5E-03	−0.56	0.98
% HLA-DR+ cells of NKG2D+ CD28− CD4-gd- T-cells	−2.48	0.20	−2.97	4.5E-03	−0.49	0.98
% HLA-DR+ cells of NKG2D+ CD4-gd- T-cells	−2.08	0.27	−2.76	4.5E-03	−0.67	0.95
% HLA-DR+ cells of NKG2D+ CD28+ CD4+ gd- T-cells	−3.35	0.034	−2.72	3.9E-03	0.63	0.95
% HLA-DR+ cells of gd+ T-cells	−1.38	0.76	−2.67	0.094	−1.28	0.91
% HLA-DR+ cells of NKG2D+ gd- T-cells	−2.08	0.26	−2.60	5.7E-03	−0.51	0.97
% HLA-DR+ cells of NKG2D+ gd + T-cells	−2.02	0.38	−2.55	0.046	−0.53	0.98
% HLA-DR+ cells of CD28− CD4− gd- T-cells	−2.60	0.20	−2.52	0.021	0.09	0.99
% HLA-DR+ cells of CD4− gd- T-cells	−2.42	0.20	−2.49	0.013	−0.07	0.99
% HLA-DR+ cells of T-cells	−2.70	0.079	−2.37	3.9E-03	0.33	0.98
% HLA-DR+ cells of NKG2D+ CD4 + gd- T-cells	−3.42	0.025	−2.20	0.016	1.23	0.85
% HLA-DR+ cells of NKG2D+ CD45RA+ CD4-gd- T-cells	−1.59	0.13	−1.49	0.013	0.10	0.99
% HLA-DR+ cells of CD45RA-CD4+ gd- T-cells	−1.61	0.13	−1.49	7.5E-03	0.12	0.99
% HLA-DR+ cells of gd- T-cells	−1.85	0.13	−1.47	0.020	0.38	0.97
% HLA-DR+ cells of CD45RA + CD4- gd- T-cells	−1.72	0.13	−1.38	0.032	0.34	0.97
% HLA-DR+ cells of NKG2D+ CD28- CD4 + gd- T-cells	−4.62	0.082	−1.20	0.59	3.42	0.39
% HLA-DR+ cells of CD28 + CD4+ gd- T-cells	−1.32	0.12	−1.14	7.5E-03	0.18	0.98
% HLA-DR+ cells of CD4+ gd- T-cells	−1.44	0.12	−1.09	0.020	0.35	0.93
% CD16+ of NK cells	−0.76	0.49	−1.07	0.068	−0.30	0.97
% IL21R+ cells of DR- CD4+ gd- T-cells	−14.56	0.018	0.43	0.97	14.99	0.050
% IL21R+ cells of CD28-CD4+ gd- T-cells	−16.00	0.025	1.04	0.90	17.03	0.050
% IL21R+ cells of CD4+ T-cells	−14.15	0.018	1.08	0.84	15.23	0.046
% CD16- cells of NK cells	0.75	0.50	1.08	0.062	0.33	0.97
% IL21R+ cells of CD45RA− CD4+ gd- T-cells	−14.14	0.018	1.09	0.84	15.22	0.046
% IL21R+ cells of CD4+ gd- T-cells	−14.33	0.018	1.14	0.84	15.46	0.046
% IL21R+ cells of CD28+ CD4+ gd- T-cells	−14.28	0.018	1.15	0.84	15.43	0.046
% IL21R+ cells of total T-cells	−13.57	0.026	1.21	0.84	14.78	0.050
% IL21R+ cells of gd- T-cells	−13.75	0.025	1.27	0.84	15.02	0.050
% IL21R+ cells of Lymphocyte	−14.42	0.019	1.29	0.84	15.71	0.046
% IL21R+ cells of CD45RA+ CD4+ gd- T-cells	−15.13	0.018	1.64	0.81	16.77	0.046
% HLA-DR- cells of CD4− gd- T-cells	2.29	0.23	2.25	0.024	−0.04	0.99
% IL21R+ cells of HLA-DR+ CD4+ gd- T-cells	−11.14	0.12	4.43	0.40	15.57	0.050

We furthered investigated whether the effect that MTX had on cell concentrations and proportions of cellular phenotypes differed significantly between responders and non-responders. Here we observed a significant difference in the changes that occurred between responders and non-responders for eleven cell phenotypes. These eleven subsets all include changes in the proportions of IL21R+ cells, usually CD4+ T-cells. Notably, this change was significant in non-responders, while unaltered in responders (Table 2, marked by red in Figure 1).

#### Changes in protein levels during MTX treatment

Treatment with MTX significantly decreased the concentration of 17 proteins in serum of responders, while no significant changes were observed in non-responders (Table 3). The regulation in the two subsets of RA patients is highly correlated (correlation between vectors of beta coefficients; *r*^2^: 0.83, *p*: 5.6*10^−6^, Figure 1B). Our data demonstrates that MTX strongly decreases the levels of IL-6, CRP, and SAA in plasma.

**Table 3:** Protein levels significantly altered during MTX treatment. Some proteins were measured using multiple separate methods.

	Change in non-responders		Change in responders	
	Beta	FDR	Beta	FDR
IL-6	−0.56	0.38	−0.99	1.7*10^−6^
CRP	−0.27	0.75	−0.88	7.6*10^−4^
CRP	−0.27	0.75	−0.88	7.6*10^−4^
SAA	−0.46	0.53	−0.78	7.6*10^−4^
CXCL10	−0.23	0.38	−0.26	7.5*10^−3^
CXCL9	−0.17	0.70	−0.34	8.6*10^−3^
CCL23 (MPIF-1)	−0.23	0.38	−0.25	8.7*10^−3^
MMP-9	−0.10	0.82	−0.26	0.012
E-Selectin	−0.11	0.55	−0.17	0.012
MCP-2	−0.36	0.36	−0.28	0.012
VEGF	0.08	0.71	−0.16	0.016
VEGF	0.08	0.71	−0.16	0.016
MMP-3	−0.01	0.93	−0.24	0.018
MMP-3	0.04	0.92	−0.29	0.018
MMP-3	−0.01	0.93	−0.23	0.019
ICAM-1	−0.03	0.87	−0.07	0.033
VCAM-1	−0.06	0.54	−0.06	0.068

#### Gene expression changes during MTX treatment

We detected significant changes of gene expression in PBMCs during treatment within the group of responders, where three genes were upregulated and 35 suppressed by MTX (Table 4; Figure 1C). These changes include the suppression of the master regulator of regulatory T-cells, FOXP3, along with several immunoglobulin genes. Gene set enrichment analysis indicated a suppression of cell cycle among RA patients responding to MTX (Table 5). Of note, these changes are beyond the changes in major cell type proportions, which we adjusted for in the analysis.

**Table 4:** Genes significantly regulated during MTX treatment among those who responded or did not respond.

		Regulation among non-responders		Regulation among responders	
Ensembl ID	HGNC	log2FC	FDR	log2FC	FDR
ENSG00000211896	IGHG1	−0.51	1.00	−1.55	4.5*10^−6^
ENSG00000211895	IGHA1	−0.65	1.00	−1.48	6.6*10^−5^
ENSG00000010319	SEMA3G	−0.83	0.74	−0.75	1.4*10^−3^
ENSG00000178445	GLDC	−0.21	1.00	−1.66	1.4*10^−3^
ENSG00000049768	FOXP3	−0.21	1.00	−0.41	1.7*10^−3^
ENSG00000211893	IGHG2	−0.54	1.00	−1.10	2.9*10^−3^
ENSG00000099250	NRP1	0.43	1.00	0.70	4.8*10^−3^
ENSG00000132465	JCHAIN	−0.42	1.00	−1.25	5.9*10^−3^
ENSG00000117399	CDC20	−0.62	1.00	−1.16	1.1*10^−2^
ENSG00000157168	NRG1	0.20	1.00	−0.54	1.3*10^−2^
ENSG00000155962	CLIC2	0.23	1.00	0.43	1.7*10^−2^
ENSG00000211679	IGLC3	0.12	1.00	−0.99	0.017
ENSG00000088325	TPX2	−0.24	1.00	−0.82	0.021
ENSG00000136235	GPNMB	0.11	1.00	0.66	0.024
ENSG00000148773	MKI67	−0.16	1.00	−0.89	0.024
ENSG00000211662	IGLV3-21	−0.75	1.00	−1.10	0.024
ENSG00000211669	IGLV3-10	−0.46	1.00	−1.25	0.024
ENSG00000211941	IGHV3-11	−0.92	1.00	−1.03	0.024
ENSG00000211663	IGLV3-19	−0.62	1.00	−1.11	0.028
ENSG00000011590	ZBTB32	−0.28	1.00	−0.48	0.028
ENSG00000211592	IGKC	−0.21	1.00	−0.99	0.030
ENSG00000115884	SDC1	−0.82	1.00	−1.76	0.036
ENSG00000126787	DLGAP5	−0.33	1.00	−1.04	0.038
ENSG00000211648	IGLV1-47	−0.40	1.00	−1.02	0.047
ENSG00000266088		−0.28	1.00	−0.41	0.060
ENSG00000211673	IGLV3-1	−0.56	1.00	−0.94	0.060
ENSG00000170476	MZB1	−0.14	1.00	−0.65	0.060
ENSG00000163599	CTLA4	−0.10	1.00	−0.33	0.061
ENSG00000211892	IGHG4	0.41	1.00	−1.24	0.062
ENSG00000239951	IGKV3-20	−0.26	1.00	−0.87	0.064
ENSG00000282122	IGHV7-4-1	−0.19	1.00	−1.23	0.064
ENSG00000211677	IGLC2	−0.36	1.00	−0.90	0.076
ENSG00000211934	IGHV1-2	−0.17	1.00	−1.03	0.082
ENSG00000211966	IGHV5-51	−0.42	1.00	−0.91	0.082
ENSG00000211644	IGLV1-51	−0.43	1.00	−0.81	0.082
ENSG00000211653	IGLV1-40	−0.60	1.00	−1.03	0.083
ENSG00000211955	IGHV3-33	−0.14	1.00	−0.88	0.096
ENSG00000211660	IGLV2-23	−0.43	1.00	−0.96	0.10
ENSG00000211706	TRBV6-1	3.58	0.07	−0.44	0.99
ENSG00000117394	SLC2A1	−0.33	0.08	0.04	0.99

**Table 5:** Gene sets suppressed during MTX treatment among RA patients who had responded to MTX treatment (EULAR classification “Moderate” or “Good”).

			By fold change		By value of *p*	
Gene set	Source	N1	AUC	FDR	AUC	FDR
PLK1 pathway	PID	43	0.62	5.5*10^−3^	0.68	8.9*10^−6^
Cyclin A B1 associated events during G2 M transition	Reactome	15	0.75	0.012	0.74	0.028
Kinesins	Reactome	21	0.71	0.012	0.71	0.028
Cell cycle mitotic	Reactome	297	0.57	0.017	0.55	0.035
Foxm1 pathway	PID	36	0.65	0.017	0.63	0.040
G1 S specific transcription	Reactome	17	0.70	0.017	0.67	0.061
Aurora B Pathway	PID	38	0.61	0.045	0.61	0.061
Aurora A Pathway	PID	29	0.62	0.072	0.62	0.028

Within non-responders only two genes were significantly altered by MTX treatment, none of which are overlapping with those significantly regulated among responders. We observed a strong increase in the expression of one of the T-cell receptor beta-chain genes, TRBV6-1 and relatively low decrease of expression of one of glucose transporter genes, SLC2A1. Gene set enrichment analysis indicated the suppression of chemokines, and Calcineurin-regulated NFAT-dependent transcription in lymphocytes (Table 6). We found no overlap between the gene set enrichments in responders and non-responders to MTX treatment. Genes regulated in responders or non-responders had slightly correlated log2 (fold changes) (*r*^2^: 0.35, *p*: 0.025).

**Table 6:** Gene sets suppressed during MTX treatment among RA patients who did not respond to MTX treatment (EULAR classification “No”).

			By fold change		By value of p	
Gene set	Source	N	AUC	FDR	AUC	FDR
Chemokine receptors bind chemokines	Reactome	40	0.71	1.0*10^−3^	0.61	0.085
NFAT TF pathway	PID	41	0.76	3.7*10^−3^	0.68	2.4*10^−3^
CD8 TCR downstream pathway	PID	52	0.75	3.7*10^−3^	0.66	6.6*10^−4^
IL12 pathway	PID	62	0.66	0.039	0.63	4.4*10^−4^

There were no genes that differed significantly in regulation among responders compared to non-responders (the two genes that were regulated by MTX in non-responders, TRBV6-1 and SLC2A1, did however have FDR <20% for having a difference in regulation in responders compared to non-responders).

### Differences between future MTX-responders and non-responders at baseline

#### Demographic and clinical factors at baseline associated to MTX response

We evaluated whether the vast set of demographic and clinical variables that were measured at baseline were associated to future MTX response. None of these variables were significantly associated to future treatment response in our cohort.

#### Cell types, cell phenotypes and protein measurements at baseline associated to later MTX response

We investigated the association between 427 immune phenotypes from flow cytometry and MTX response but did not detect any significant differences between future responders and non-responders at baseline.

We investigated the association of 51 proteins measured in MTX naïve samples (whereof 16 were investigated using multiple assays) and MTX response. No association reached an FDR below 10%.

#### Gene expression levels at baseline associated to future MTX response

There were 88 genes for which expression levels at baseline were significantly different between patients who would later respond to MTX, compared to those who would not (Supplementary Table S2). However, none of these 88 genes remained significant in every of the 60 LOO iteration. The maximum number of leave-one-out iterations when a gene was significant was 58 (for 8 genes). The number of significant genes across the 60 leave-one-out iterations fluctuated between zero and 1,066. We therefore concluded that no single gene expression level at baseline was consistently associated to future clinical response. Further, given the large fluctuation in analysis results depending on the exclusion of single samples in these cross-sectional analyses, we also refrained from performing gene set enrichment analysis.

#### Predicting MTX response

**Figure 2:**
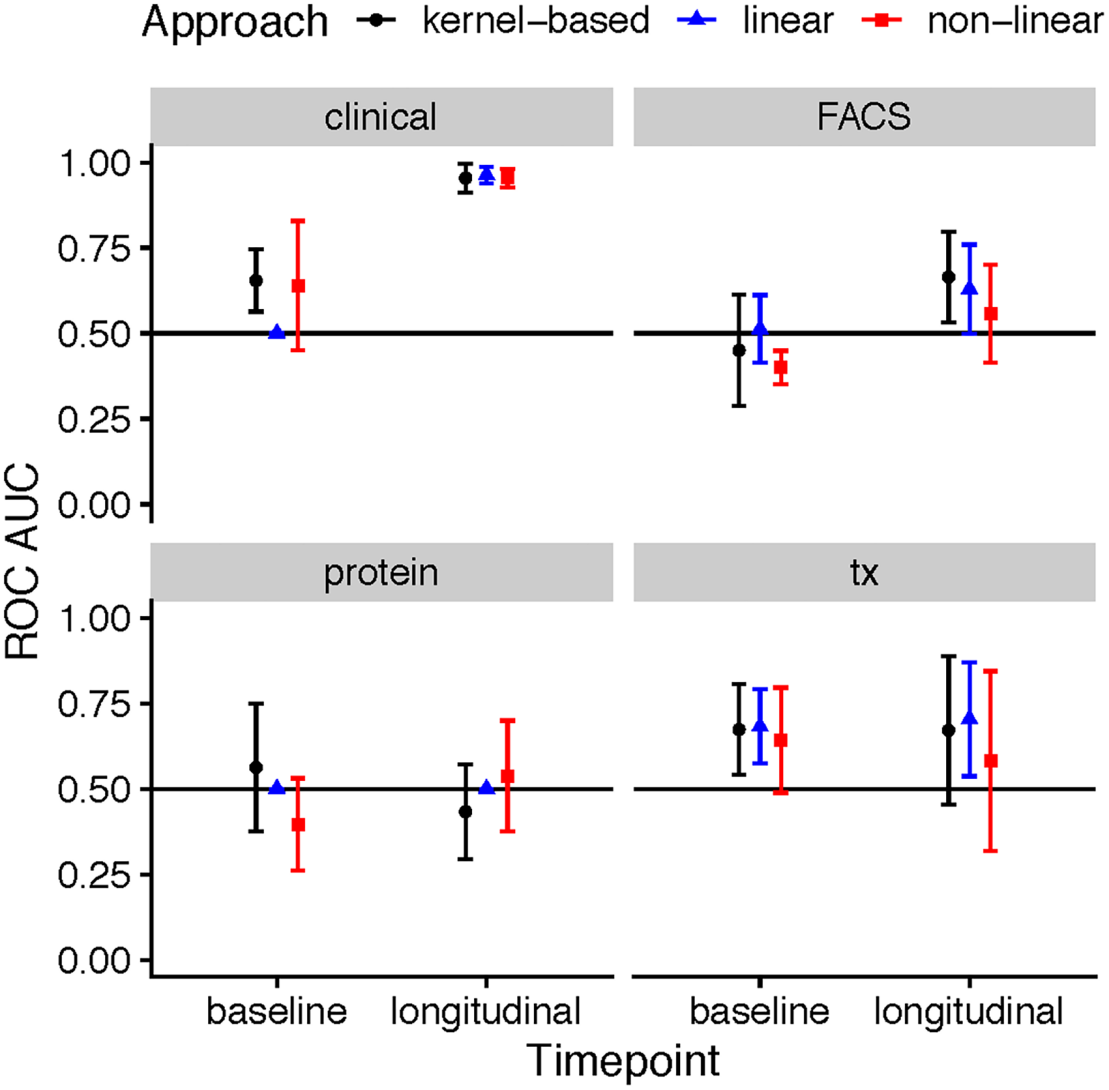
ROC AUC performance of ML models predicting response based on clinical, flow cytometry, protein, and RNA-seq data in MTX therapies recorded during the baseline patient visit, and a longitudinal difference between the three-month follow-up and baseline. The points in the plots showcase the mean ROC AUC values of the median repeat in each of the ML tasks, along with the corresponding 95% confidence interval of the standard error of the mean (SEM).

The predictive ability varied across the four data types (clinical, flow cytometry, transcriptomics, and protein), time point, and employed method. At baseline, a total of three combinations achieved ROC AUCs with a confidence interval which did not include the 0.5 level: the kernel-based prediction model utilizing clinical variables (mean AUC: 0.65, 95% CI: 0.56–0.75), the kernel-based and linear models of gene expression data (mean AUC: 0.67, 95% CI: 0.54–0.81 and mean AUC: 0.68, 95% CI: 0.57–0.79, respectively) (Figure 2). As expected, the longitudinal changes in clinical variables resulted in a very accurate prediction of response. Further, changes in measurements of gene expression, protein or flow cytometry phenotypes did not achieve successful prediction models. Further, although baseline FACS models yielded no successful predictions, the longitudinal kernel-based model resulted in more predictive models with mean AUC of 0.66, 95% CI: 0.53–0.80; in transcriptomics, the linear longitudinal model displayed a positive predictivity with a mean ROC AUC of 0.70, with a 95% CI of 0.54–0.87.

## Discussion

Here we report insights from a clinically and biologically well-characterized cohort of newly diagnosed RA patients starting MTX treatment and followed during 3 months of treatment. The availability of information regarding important demographic, clinical and immunological confounders enabled us to conduct a thorough and robust analysis, less sensitive to bias. We detect strong effects of MTX across clinical measurements, protein expression in peripheral blood, gene expression in PBMCs and cell phenotype proportions, mainly a suppression across all tissue types. The biological effects in immune cell proportions and gene expression differed between responders and non-responders, indicating that there are indeed different biological processes occurring in those who respond clinically compared to those who do not.

At the rheumatology clinics the rheumatologist evaluates the treatment response of each patient according to their specific disease phenotype and progression, and one treatment response definition might not fit all patients. This can also be seen in the literature where different investigators use a diverse set of treatment outcomes. We chose to analyze EULAR treatment response that takes both the resulting DAS28 and the change induced by treatment into account, which is often employed in clinical trials. In this study we chose to analyze whether patients had any convincing effect of treatment, where a moderate and good EULAR treatment response were merged. We focused on the statistically most powerful analyses: the effect of treatment within individuals. In this analysis, we were particularly interested in whether different mechanisms are active in the patients that respond to MTX, compared to those who do not respond. Secondly, we also attempted to detect biomarkers that can predict whether a patient will respond to MTX treatment by using measurements from the baseline visit only.

In our flow cytometry data of PBMC we observed significant changes in many immune cell proportions during MTX treatment. MTX strongly diminished the proportion of HLA-DR+ T-cells in relation to other T cells in responders and non-responders, but more notably so within responders. As HLA-DR expression is considered a sign of activated T-cells, this indicates that across all patients MTX was able to specifically suppress the activation of T-cells, alternatively affect their abundance in peripheral blood. Additionally, the proportion of T-cells expressing IL21R were suppressed exclusively in non-responders. IL21 is a pleiotropic cytokine with context-dependent mainly pro-inflammatory effects on T cell differentiation (11), and thus a potential treatment target in RA (12). The receptor for IL21 is expressed upon cellular activation on T cells as well as on many other leukocytes. We analyzed IL21R expression on T cells as well as on NK cells, B cells and monocytes, and found suppressed proportions of only T cells expressing IL21R. The decreased proportion of IL-21-expressing T-cells in non-responding patients could indicate an altered tissue distribution of these cells, or a reduced overall expression of IL21R in T cells specifically.

The effect of MTX on protein levels in serum was similar across responders and non-responders. However, the coefficients of change were always larger for responders, indicating that the detected regulation tended to be stronger among responders. As expected we saw a drop in CRP levels. Blockage of IL-6 has been shown to be clinically beneficial in RA (reviewed in (13)), and here we observed a significant decrease in IL-6 among patients who responded to treatment. We did not observe a significant regulation of IL-6 gene expression, which might be due to limitations in power or a decreasing of IL-6 gene expression by cells other than PBMC. In addition, VEGF was significantly decreased by MTX within responders, whereas levels in non-responders increased slightly (not significant). Here the difference in regulation among responders and non-responders was nominally significant (*p*: 0.011). VEGF has previously been suggested to be positively correlated with disease severity (DAS28) and CRP levels (14). In our material there was a positive correlation between CRP and VEGF among treatment naïve patients (*r*^2^: 0.44, *p*: 2.4*10^−5^), but no correlation was seen after MTX treatment (*r*^2^: 0.01, *p*: 0.92). CCL23 has been suggested as a severity marker for RA (15), and we observed that it decreased significantly during treatment in responders. We saw a significant correlation between CCL23 and DAS28 in MTX-naïve patients (*r*^2^: 0.27, *p*: 0.0086), but this correlation disappeared in MTX-treated patients (*r*^2^: 0.02, *p*: 0.85). We also found a suppression of CXCL10 and E-selectin, in line with data in a previous reports (16, 17). Additionally, we detected the suppression of pro-inflammatory proteins Serum Amyloid A and CXCL9, and the metalloproteinase MMP-9 in responders.

MTX treatment had a large effect on gene expression levels, with some similarities between responders and non-responder, i.e., no single genes had a significantly differential regulation in responders as compared to non-responders. Among the patients that responded to MTX there was a clear suppression of the expression of cell cycle genes, indicating that MTX decreased the proliferation of the PBMC alternatively that MTX caused sequestration of proliferating cells in tissues. Also, the expression of several immunoglobulin genes were significantly suppressed by MTX among the responders. In our flow cytometry panel we measured the proportion of IgD-CD138+ CD27+ of all B cells, a staining that is considered to identify antibody-secreting plasma cells. The proportion of plasma cells measured this way was indeed suppressed by MTX, but had a FDR > 10% (beta: −033, *p*: 0.015). These findings could be a result of an altered distribution of immunoglobulin-expressing cells in the body induced by MTX, or a direct effect of MTX on antibody-production and B cell maturation processes. The gene expression level for the master regulator of regulatory T-cells, FOXP3, was also suppressed by treatment in those who responded to MTX. This was surprising given several earlier reports indicating that successful MTX treatment increases the proportion of regulatory T-cells in the peripheral blood of individuals with RA (18). Notably, transcripts of FOXP3 is expressed not only by regulatory T-cells, but also transiently by activated T-cells (19) and other cell lineages (20).

Among non-responders the effect of MTX on gene expression was generally weaker than in responders. The two genes that were significantly regulated among non-responders only, TRBV6-1 (log2FC:3.58) and SLC2A1 (log2FC: −0.33), were the genes with the strongest evidence for differential regulation by MTX treatment in responders compared to non-responders (FDR < 20%). Comparing the log2 (fold changes) in responders and non-responders showed only weak correlation (*r*^2^: 0.35, *p*: 0.025). Notably, gene set enrichment analysis indicated the suppression of inflammatory gene sets such as chemokines and chemokine receptors, IL12, NFAT and the pathway downstream the TCR of CD8+ T-cells also within clinical non-responders, gene sets which were not enriched in responders. Non-responders also experienced a decrease in the proportion of IL21R+ T-cell subsets during treatment, and an increased expression of the T-cell receptor gene TRBV6-1.

Prednisolone has a large effect on immune cells (21). In exploratory analysis, we accordingly observed that the effect of MTX and prednisolone jointly was larger than the effect of MTX alone on gene expression (results not shown). This exemplifies why adjusting for prednisolone in our analyses was essential.

We did not detect any biomarkers or prediction models with sufficient predictive value to aid in MTX therapy at baseline. Although the transcriptome models displayed positive predictivity, the ROC AUCs were relatively low which indicates that these results are fit as supplementary evidence, rather than serve as a basis of treatment choice. The set of proteins we investigated was, however, rather limited (n:51). We assayed a broad range of immune phenotypes, but the focus was to look at all major cell types and not the more functional ones. Previous reports have indicated that non-responders of MTX had a higher concentration of monocytes, and a proportion of CD14brightCD16−, CD14brightCD16+ and CD14dimCD16+ monocyte subsets before treatment initiation (22). Here neither of those signals was replicated, although we do detect a trend for higher proportion of CD14brightCD16+ among non-responders (OR: 0.86, *p*: 0.096). The level of IL1beta produced by PBMC has previously been suggested as a biomarker of response to MTX (23) but we detect no such pattern in serum, and when looking at gene expression by PBMC the level was slightly lower in future responders but far from significant [log2 (fold change): −0.34, *p*: 0.22]. Our results indicate that no major immune cell phenotypes in peripheral blood was able to predict who will later respond to MTX. Plant et al. (24) has previously demonstrated similar predictive ability of whole blood gene expression at baseline as we observe here. They additionally show that the difference in gene expression at baseline and 4 weeks into treatment is valuable to predict long-term MTX response. We are unable to interrogate this in our sample set as patients did not donate blood until their follow up clinical appointment at 3 months. Overall, we demonstrate some prediction models that are significantly better than random, yet not strong enough to warrant clinical implementation. This might be due to the heterogeneity, and limited size, of the included sample set.

In this study we are focusing on measurements done in peripheral blood due to the accessibility and low discomfort for the donors. This might however limit our possibility of detecting biomarkers or understanding the mechanisms associated to a good response to MTX. The important processes might in fact happen elsewhere in the body, as in the synovial or lymphoid tissue. Another limitation of this project is that gene expression alterations in specific cell types might be diluted and missed when total RNA is sequenced. We only investigated a subset of all cell type proportions and proteins, so important biomarkers might have been missed.

## Conclusion

In summary, we herein show that MTX treatment leads to significantly different biological effects among those who respond clinically to treatment and those who do not. Within those who responded to MTX we observed a suppression of the proportions of HLA-DR+ T-cell subsets, a suppression of cell cycle genes, and a downregulation of IL-6. In non-responders we instead observed the suppression of IL21R+ T-cell subsets. These findings might represent biological processes that are involved in the clinical response, or lack of response, to MTX among RA patients.

## Data availability statement

The datasets generated and analysed in the current study is not available in a public repository since this is restricted by GDPR and the ethical permissions for COMBINE. We encourage researchers with an interested in the material to arrange an ethical approval and contact author LB with data requests for applicable studies.

## Ethics statement

The studies involving human participants were reviewed and approved by the Stockholm (number 2010-351-31-2) and Uppsala (2009-013) Regional Ethics Committees. The patients/participants provided their written informed consent to participate in this study. Written informed consent was obtained from the individual(s) for the publication of any potentially identifiable images or data included in this article.

## Funding

The vast majority of expenses related to this study including laboratory measurements and study costs were funded by the pharmaceutical company Novo Nordisk A/S. Novo Nordisk was involved in the design and collection of data. Pfizer Inc. contributed funds for conducting the included analyzes. Pfizer Inc was involved in analysis design, decision to publish and preparation of the manuscript. LP was supported by NORA consortium.

## Author contributions

BB formulated the research question, derived a subset of the included variables, designed and performed the statistical analysis, interpreted the results and wrote the manuscript. NY trimmed and aligned RNA seq data, participated in analysis design and manuscript reviewing. TU reviewed and cleaned the flow cytometry data, and performed statistical analysis of the flow cytometry data. DZ participated in analysis design and interpretation, as well as illustrations and paper manuscript review. MMa designed, performed and interpreted the machine learning analysis. LF cleaned and integrated all the different data sets. HW, LP, and SJ participated in results interpretation and manuscript review. NP, MMü, and PS built the biobank, ran the flow cytometry and analysed the flow cytometry raw data. AH and AC selected the patient and performed the clinical evaluation. LK and AC initiated the COMBINE study. LB designed the patient sample laboratory analyses, supervised the laboratory work, coordinated data collection and interpreted flow cytometry and plasma protein findings. All authors contributed to the article and approved the submitted version.

## Conflict of interest

SJ, MMa, and DZ were employed by Pfizer. LF was employed by Novo Nordisk A/S during data generation for this study and currently is employed by Nucleus Genomics.

The remaining authors declare that the research was conducted in the absence of any commercial or financial relationships that could be construed as a potential conflict of interest.

## Publisher’s note

All claims expressed in this article are solely those of the authors and do not necessarily represent those of their affiliated organizations, or those of the publisher, the editors and the reviewers. Any product that may be evaluated in this article, or claim that may be made by its manufacturer, is not guaranteed or endorsed by the publisher.

## Footnotes

^1^http://www.pathway.jp

^2^http://cgap.nci.nih.gov/Pathways/BioCarta_Pathways

^3^http://stke.sciencemag.org/about/help/cm

^4^http://www.sigmaaldrich.com/life-science.html

^5^http://www.signaling-gateway.org

^6^http://www.sabiosciences.com/ArrayList.php

^7^http://pid.nci.nih.gov

^8^http://www.reactome.org

^9^http://matrisomeproject.mit.edu

## Abbreviations

ACPA: Anti-citrullinated protein antibodies
MTX: Methotrexate
RA: Rheumatoid arthritis
PCA: Principal components analysis
PBMC: Peripheral blood mononuclear cells
EULAR: The European Alliance of Associations for Rheumatology
HLA-DR: Human leukocyte antigen – DR isotype
CRP: C-reactive protein
DAS28: Disease activity score 28 joints
CCP2: Second generation anti-cyclic citrullinated peptide
RNA: Ribonucleic acid
UPPMAX: Uppsala Multidisciplinary Center for Advanced Computational Science
LOO: Leave one out
AUC: Are under curve
ROC: Receiver operator characteristic
