# Supplementary material for: Molecular signature of methotrexate response among rheumatoid arthritis patients
Source: Front Med (Lausanne). 2023 Mar 27;10:1146353. doi: 10.3389/fmed.2023.1146353 (PMC10084884; doi:10.3389/fmed.2023.1146353)
Supplement: Supplementary file 1 [file Table_1.DOCX]

## **Supplementary Tables**

Table S1. MTX has a stronger disease modifying effect in those who responded to treatment. Coefficients and p-values from a mixed linear model (clinical phenotype ~ visit*response01_ESR + Prednisolone + I(pat_id)), modelled using *lme* (*nlme* v. 3.1-137) and *emmeans* (v. 1.3.3). Findings with a p-value <= 0.05 are marked in bold.

|  | Non-responders | | Responders | | Difference | |
| --- | --- | --- | --- | --- | --- | --- |
| Clinical phenotype | beta | p | beta | p | beta | p |
| ESR | -1.26 | 0.68 | **-9.97** | **2.7*10^-6^** | **-8.71** | **0.015** |
| CRP | -2.62 | 0.49 | **-7.00** | **5.5*10^-3^** | -4.38 | 0.32 |
| DAS28 | -0.47 | 0.02 | **-2.08** | **1.4*10^-25^** | **-1.61** | **2.4*10^-9^** |
| Swollen joints | **-4.48** | **1.7*10^-4^** | -6.67 | 1.8*10^-14^ | -2.19 | 0.099 |
| Tender joints | -1.55 | 0.11 | **-5.52** | **9.9*10^-14^** | **-3.98** | **5.7*10^-4^** |
| Pain (VAS mm) | **-20.55** | **7.9*10^-4^** | **-26.25** | **3.3*10^-10^** | -5.71 | 0.40 |
| Physical function (HAQ) | -0.12 | 0.28 | **-0.47** | **3.6*10^-9^** | **-0.35** | **6.8*10^-3^** |
| Patient assessment of global status | **-13.05** | **0.039** | **-22.39** | **2.1*10^-7^** | -9.34 | 0.20 |
| Health professional assessment of global status | -8.32 | 0.061 | **-37.31** | **1.2*10^-21^** | **-28.99** | **2.2*10^-7^** |

Table S2. Genes that were differently expressed between future responders compared to non-responders before initiating MTX treatment. Log2FC = log2(fold change), FDR = false discovery rate, LOO iteration significant = number of leave-one-out iterations (out of 60 total) where the gene had an FDR < 0.1.

| Ensembl ID | HGNC | log2FC | p-value | FDR | LOO iterations significant |
| --- | --- | --- | --- | --- | --- |
| ENSG00000138166 | DUSP5 | -0.73 | 8.4E-06 | 0.037 | 58 |
| ENSG00000149131 | SERPING1 | -1.69 | 7.8E-06 | 0.037 | 58 |
| ENSG00000020577 | SAMD4A | -0.85 | 4.9E-06 | 0.037 | 58 |
| ENSG00000198133 | TMEM229B | -0.54 | 5.6E-06 | 0.037 | 58 |
| ENSG00000271795 |  | 0.99 | 1.5E-05 | 0.045 | 56 |
| ENSG00000226688 | ENTPD1-AS1 | 0.67 | 1.8E-05 | 0.045 | 58 |
| ENSG00000130487 | KLHDC7B | -0.78 | 1.6E-05 | 0.045 | 58 |
| ENSG00000175283 | DOLK | -0.46 | 2.1E-05 | 0.046 | 57 |
| ENSG00000228223 | HCG11 | 0.46 | 2.9E-05 | 0.056 | 57 |
| ENSG00000232725 |  | 1.41 | 3.2E-05 | 0.056 | 55 |
| ENSG00000225492 | GBP1P1 | -1.37 | 4.0E-05 | 0.059 | 54 |
| ENSG00000162772 | ATF3 | -0.97 | 5.1E-05 | 0.059 | 57 |
| ENSG00000124762 | CDKN1A | -0.69 | 4.9E-05 | 0.059 | 58 |
| ENSG00000085644 | ZNF213 | -0.40 | 4.9E-05 | 0.059 | 55 |
| ENSG00000108679 | LGALS3BP | -0.77 | 4.3E-05 | 0.059 | 57 |
| ENSG00000188290 | HES4 | -1.27 | 6.6E-05 | 0.062 | 58 |
| ENSG00000228037 |  | -0.82 | 5.7E-05 | 0.062 | 54 |
| ENSG00000206559 | ZCWPW2 | 1.69 | 6.7E-05 | 0.062 | 49 |
| ENSG00000251192 | ZNF674 | 0.47 | 6.4E-05 | 0.062 | 56 |
| ENSG00000097021 | ACOT7 | -0.51 | 9.7E-05 | 0.068 | 55 |
| ENSG00000158748 | HTR6 | 1.70 | 1.0E-04 | 0.068 | 52 |
| ENSG00000162654 | GBP4 | -0.56 | 1.0E-04 | 0.068 | 56 |
| ENSG00000228506 |  | 0.88 | 1.1E-04 | 0.068 | 53 |
| ENSG00000160932 | LY6E | -0.90 | 8.3E-05 | 0.068 | 57 |
| ENSG00000152766 | ANKRD22 | -1.21 | 1.1E-04 | 0.068 | 55 |
| ENSG00000225131 | PSME2P2 | -0.98 | 8.0E-05 | 0.068 | 55 |
| ENSG00000109066 | TMEM104 | -0.37 | 1.1E-04 | 0.068 | 54 |
| ENSG00000170677 | SOCS6 | 0.59 | 1.0E-04 | 0.068 | 55 |
| ENSG00000157601 | MX1 | -1.09 | 1.1E-04 | 0.068 | 53 |
| ENSG00000108798 | ABI3 | -0.50 | 1.2E-04 | 0.069 | 54 |
| ENSG00000138642 | HERC6 | -0.69 | 1.4E-04 | 0.076 | 56 |
| ENSG00000088827 | SIGLEC1 | -1.46 | 1.4E-04 | 0.076 | 56 |
| ENSG00000261098 |  | 0.65 | 1.4E-04 | 0.076 | 53 |
| ENSG00000236200 | KDM4A-AS1 | 0.86 | 1.6E-04 | 0.076 | 53 |
| ENSG00000277007 |  | 1.07 | 1.6E-04 | 0.076 | 51 |
| ENSG00000167081 | PBX3 | 0.29 | 1.6E-04 | 0.076 | 53 |
| ENSG00000140464 | PML | -0.44 | 1.6E-04 | 0.076 | 55 |
| ENSG00000196839 | ADA | -0.44 | 1.6E-04 | 0.076 | 54 |
| ENSG00000126709 | IFI6 | -1.30 | 1.9E-04 | 0.077 | 55 |
| ENSG00000230979 |  | -1.10 | 2.1E-04 | 0.077 | 46 |
| ENSG00000198039 | ZNF273 | 0.49 | 1.9E-04 | 0.077 | 52 |
| ENSG00000148935 | GAS2 | 0.99 | 2.1E-04 | 0.077 | 52 |
| ENSG00000110514 | MADD | -0.27 | 1.7E-04 | 0.077 | 52 |
| ENSG00000185864 | NPIPB4 | 0.82 | 1.9E-04 | 0.077 | 53 |
| ENSG00000108691 | CCL2 | -1.96 | 2.0E-04 | 0.077 | 52 |
| ENSG00000068079 | IFI35 | -0.64 | 2.1E-04 | 0.077 | 52 |
| ENSG00000125730 | C3 | -0.91 | 2.0E-04 | 0.077 | 53 |
| ENSG00000128203 | ASPHD2 | -0.43 | 1.8E-04 | 0.077 | 53 |
| ENSG00000140650 | PMM2 | 0.68 | 2.2E-04 | 0.077 | 51 |
| ENSG00000173786 | CNP | -0.32 | 2.2E-04 | 0.077 | 52 |
| ENSG00000149798 | CDC42EP2 | -0.93 | 2.3E-04 | 0.078 | 52 |
| ENSG00000187608 | ISG15 | -1.31 | 2.7E-04 | 0.080 | 46 |
| ENSG00000162694 | EXTL2 | 0.49 | 2.6E-04 | 0.080 | 49 |
| ENSG00000257226 |  | 1.72 | 2.7E-04 | 0.080 | 48 |
| ENSG00000138801 | PAPSS1 | 0.40 | 2.7E-04 | 0.080 | 46 |
| ENSG00000127399 | LRRC61 | -0.56 | 2.5E-04 | 0.080 | 47 |
| ENSG00000272849 |  | 0.98 | 2.5E-04 | 0.080 | 48 |
| ENSG00000140463 | BBS4 | 0.24 | 2.5E-04 | 0.080 | 46 |
| ENSG00000178773 | CPNE7 | -1.27 | 2.6E-04 | 0.080 | 45 |
| ENSG00000171954 | CYP4F22 | -1.06 | 2.5E-04 | 0.080 | 50 |
| ENSG00000186810 | CXCR3 | -0.62 | 2.8E-04 | 0.082 | 45 |
| ENSG00000179889 | PDXDC1 | 0.74 | 2.9E-04 | 0.082 | 45 |
| ENSG00000134321 | RSAD2 | -1.55 | 3.0E-04 | 0.083 | 49 |
| ENSG00000167208 | SNX20 | -0.48 | 3.0E-04 | 0.083 | 43 |
| ENSG00000115155 | OTOF | -1.38 | 3.2E-04 | 0.086 | 41 |
| ENSG00000140105 | WARS | -0.53 | 3.2E-04 | 0.086 | 39 |
| ENSG00000204516 | MICB | 1.26 | 3.3E-04 | 0.088 | 39 |
| ENSG00000148175 | STOM | -0.40 | 3.6E-04 | 0.090 | 36 |
| ENSG00000142089 | IFITM3 | -1.41 | 3.6E-04 | 0.090 | 36 |
| ENSG00000204556 |  | 1.07 | 3.6E-04 | 0.090 | 36 |
| ENSG00000134575 | ACP2 | -0.52 | 3.7E-04 | 0.091 | 36 |
| ENSG00000234678 | ELF3-AS1 | 1.13 | 3.8E-04 | 0.092 | 32 |
| ENSG00000121671 | CRY2 | -0.24 | 4.0E-04 | 0.092 | 28 |
| ENSG00000137486 | ARRB1 | -0.39 | 3.9E-04 | 0.092 | 31 |
| ENSG00000170855 | TRIAP1 | -0.35 | 4.0E-04 | 0.092 | 32 |
| ENSG00000131969 | ABHD12B | 0.84 | 4.0E-04 | 0.092 | 30 |
| ENSG00000261997 |  | 1.08 | 3.9E-04 | 0.092 | 34 |
| ENSG00000143412 | ANXA9 | 0.68 | 4.2E-04 | 0.093 | 32 |
| ENSG00000180769 | WDFY3-AS2 | 1.15 | 4.2E-04 | 0.093 | 31 |
| ENSG00000232593 | KANTR | 0.49 | 4.2E-04 | 0.093 | 28 |
| ENSG00000171161 | ZNF672 | -0.37 | 4.4E-04 | 0.094 | 22 |
| ENSG00000114315 | HES1 | -0.96 | 4.4E-04 | 0.094 | 25 |
| ENSG00000259248 | USP3-AS1 | 0.99 | 4.4E-04 | 0.094 | 25 |
| ENSG00000059378 | PARP12 | -0.45 | 4.6E-04 | 0.095 | 25 |
| ENSG00000244687 | UBE2V1 | 0.62 | 4.6E-04 | 0.095 | 22 |
| ENSG00000197122 | SRC | -0.46 | 4.7E-04 | 0.096 | 25 |
| ENSG00000214872 | SMTNL1 | -0.78 | 4.8E-04 | 0.098 | 21 |
| ENSG00000142961 | MOB3C | -0.29 | 4.9E-04 | 0.098 | 21 |
