# Supplementary material for: Molecular signature of methotrexate response among rheumatoid arthritis patients
Source: Front Med (Lausanne). 2023 Mar 27;10:1146353. doi: 10.3389/fmed.2023.1146353 (PMC10084884; doi:10.3389/fmed.2023.1146353)

Supplementary figure 1. PBMC or lysed whole blood were stained by fluorescently labeled antibodies and analysed by flow cytometry. B cells, T cells, NK cells, monocytes and granulocytes, as well as subpopulations of these major celltypes, were identified by a gating strategy shown in this figure.

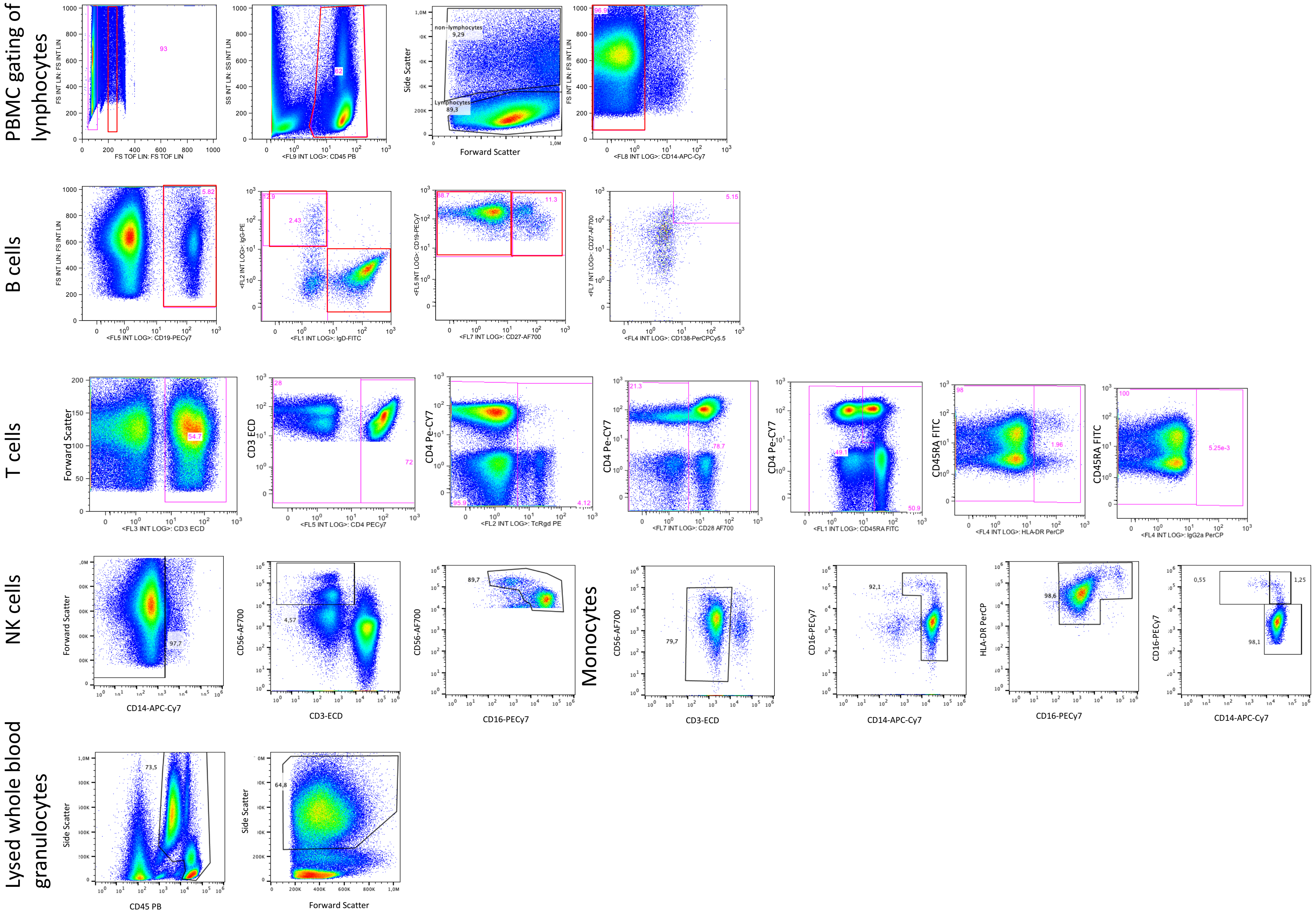

Supplement: Supplementary file 2 [file Image_1.pdf]
